# Supplementary material for: MetaRibo-Seq measures translation in microbiomes
Source: Nat Commun. 2020 Jun 29;11:3268. doi: 10.1038/s41467-020-17081-z (PMC7324362; doi:10.1038/s41467-020-17081-z)
Supplement: Supplementary file 10 — Supplementary Data 7 [file 41467_2020_17081_MOESM10_ESM.zip › File2/Confidence_High_Taxonomy/395321_out.krona.html]

Javascript must be enabled to view this page.

members
magnitude
magnitudeUnassigned
count
unassigned
taxon
rank

395321\_out

10

superkingdom
10
2

phylum
1239
10

class
186801
10

186802
10
order

541000
10
family

1263
10
genus

46228
10
species

SRS012273\_contig\_number\_33164SRS015663\_contig\_number\_16789SRS019685\_contig\_number\_10344SRS050752\_contig\_number\_24009SRS051031\_contig\_number\_13527SRS062701\_contig\_number\_5228SRS098571\_contig\_number\_3328SRS142542\_contig\_number\_22704SRS143181\_contig\_number\_13612SRS147766\_contig\_number\_10438
